# Congenital Long QT Syndrome (LQTS) in Infancy: A Challenging Case

**DOI:** 10.7759/cureus.51810

**Published:** 2024-01-07

**Authors:** Mohammed Aldirawi, Rehab Musa, Moataz Hamdi, Lemis Yavuz

**Affiliations:** 1 Pediatrics, Al Jalila Children’s Hospital, Dubai, ARE

**Keywords:** unexplained syncope, sudden early death, cardiac arrythmia, congenital long qt syndrome, brief resolved unexplained events

## Abstract

Long QT syndrome (LQTS), is an arrhythmia disorder, related to ventricular myocardial repolarization characterized by a prolonged QT interval on the electrocardiogram that can lead to symptomatic ventricular arrhythmias and increase the mortality rate. The prevalence of congenital LQTS is about 1 in 2000 live births.

Here, we report the case of a two-month-old female, with a significant family history of early death, who was brought to our emergency with an episode of blueish discoloration. The initial workup was positive for COVID-19 in the respiratory panel, so she was admitted as a case of bronchiolitis. It was a challenge because of the overlapping presentation between a serious condition and other common pediatric illnesses.

Furthermore, the paper aims to increase awareness of this condition among physicians and emphasizes the importance of obtaining a complete medical history, physical examination, and family screening in selected cases to facilitate early diagnosis and timely management.

## Introduction

The diagnosis of congenital long QT syndrome (LQTS) was mentioned last century in 1957 when Jervell and Lange-Nielsen first published an article about four young siblings having a combination of congenital deafness and peculiar heart disease with syncopal episodes and obvious prolongation of the QT interval in the electrocardiograms (ECGs) [1].

In LQTS, two clinical phenotypes have been described, a common autosomal dominant form, originally named the Romano-Ward syndrome, a purely cardiac phenotype of QT prolongation, and a rare autosomal recessive form, originally named the Jervell and Lange-Nielsen syndrome, that is associated with sensorineural deafness [2].

LQTS can lead to sudden cardiac death (SCD), yet these syndromes are often asymptomatic and clinically undetected, despite the prolongation of the QT interval [3].

This highlights the critical importance of a timely diagnosis and management in such patients, even in asymptomatic patients, particularly genetic counseling with each life stage [3].

## Case presentation

The two-month-old female was brought to our emergency department with a history of frothy secretions from the Infant's mouth and nose while she was sleeping. This episode happened three hours after the last feed. Her father noticed the cold extremities and bluish face for which he started chest thrusts. There was no history of coughing or choking, but only a runny nose for two days. The review of the other systems was unremarkable.

The infant is a preterm baby at 32 weeks due to fatal distress; her birth weight was 1.8 kg to a healthy unrelated parent. She has three other preterm siblings; one of them died at the age of 80 days because of an unexplained event that, at the post-mortem, was diagnosed as cardiac failure. The other two siblings were healthy.

In the emergency, the infant was irritable with borderline saturation at 90%. Capillary refill time was two seconds with mottled skin. She was placed on 2L/minutes of oxygen nasal cannula and saturation improved to 95%. The respiratory rate was 40 per minute, the heart rate was 170 per minute, and the blood pressure was within normal limits for her age. Chest and heart examination revealed fine crackles and rhonchi with no appreciated murmur.

**Figure 1:**
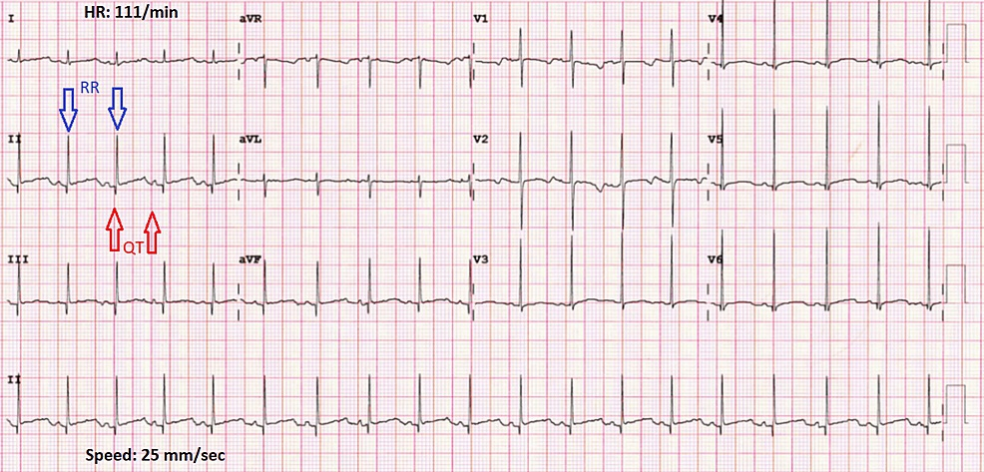
Corrected QT interval (QTc)

Blood gases, full blood count, C-reactive protein, and procalcitonin were normal, and COVID-19 polymerase chain reaction (PCR) was positive. Hence she was admitted for bronchiolitis due to COVID-19. She was placed on a continuous monitor. Reassessment showed recurrent episodes of desaturation reached 70% while the baby was on oxygen. However, a chest examination did not show significant changes to explain these episodes. Reviewing the monitor showed an association between the desaturation and bradycardia. ECG was done and showed prolonged QT interval, corrected QT interval (QTc) 544 ms (Figure 1). An echocardiogram showed normal heart structure and function. EEG didn’t show any abnormal changes and the brain ultrasound scans (USS) were normal. Hearing tests and ophthalmological evaluations were normal.

The patient was started on oral propranolol 2.5 mg, four times daily. The parents passed cardiopulmonary resuscitation training and she was discharged home safely with stable vitals and no episodes of bradycardia or oxygen desaturations.

Given the family history of deaths and the prolonged QT interval, sequencing of the coding regions and splice sites of the 38 genes associated with long QT condition was done and identified two heterozygous variants of uncertain significance in KCNQ1 and CACNB2. Family members screening with ECG was normal and the screening with genetic testing was recommended.

Our patient makes regular cardiology clinic follow-ups. The QT interval improved (QTc: 485 ms). Exercise-stressed ECG is planned for the future.

## Discussion

Prolonged QT interval is a rare fatal disease especially in infancy [4]. The signs and symptoms vary from mild palpitation to sudden death. It can easily be missed, especially when it comes to other symptoms, and for that, it is a challenging disease. Prolonged QT interval could be primary or secondary. Even with the high suspicion of congenital LQTS, investigations for acquired long QT intervals and aggravating factors should be investigated [1].

We describe a case of a life-threatening event that was not explained by mild respiratory symptoms even with the positive COVID-19 PCR. The workup involved assessing one of the most common diseases in infancy which was bronchiolitis. The COVID-19 infection would explain the initial presentation of a runny nose and bluish discoloration. However, the baby continued to have episodes of desaturation and bradycardia despite being on oxygen and without any deterioration on chest examination. Hence, more investigations were done, and ECG revealed prolonged QT interval when cardiology became involved.

Detailed history and focused physical examination are the key parts to reaching a proper diagnosis. The red flags in our case were the cold extremities, irritability, desaturation with bradycardia, and the family history of sudden infant death which may have been due to LQTS as seen in about 5% to 10% of the cases [5]. The EEG and brain USS were also done to exclude conditions that could have caused the desaturation, such as observed during a convulsion.

The main tool to arrive at a diagnosis is ECG, the 24-hour ambulatory ECG monitoring is performed as well, looking for arrhythmias or any dynamic T wave changes. A normal ECG is not enough to exclude LQTS among families with genetically proven/confirmed LQTS [6]. A bicycle or treadmill stress test needs to be done if the patient is old and looking for exercise-associated arrhythmias [1].

Calculation of the LQTS diagnostic score, also known as the Schwartz score (Table 1), can be used to assess the probability of LQTS [7].

**Table 1:** Schwartz score diagnostic criteria for long QT syndrome (LQTS) QTc: Corrected QT interval; LQTS: Long QT syndrome

	Points
- Electrocardiographic findings	
A. QTc	
≥480 ms	3
460 ms to 479 ms	2
450 ms to 459 ms (in males)	1
B. QTc fourth minute of recovery from exercise stress test ≥480 ms	1
C. Torsades de pointes	2
D. T wave alternans	1
E. Notched T wave in three leads	1
F. Low heart rate for age	0.5
- Clinical history	
A. Syncope	
With stress	2
Without stress	1
B. Congenital deafness	0.5
- Family history	
A. Family members with definite LQTS	1
B. Unexplained sudden cardiac death below age 30 among immediate family members	0.5

The QTc is calculated by dividing the QT interval by the square root of the preceding RR interval (QTc = QT interval ÷ √RR interval (in sec)) and the most normal reference ranges are based upon measurements from lead II and lead V5 [7]. The probability of having LQTS is low for 1 point or less, intermediate for 1.5 to 3 points, and high for ≥3.5 points [7]. In our case, the probability of LQTS was high as the score was 4.5 points while there were no arrhythmias which could be ventricular tachyarrhythmias in about 16% of the cases [8].

Genetic testing should be done for all patients with suspicion of congenital LQTS based on the medical history, ECG findings, and results of any additional testing, such as a high or an intermediate Schwartz score [9].

For any patient with a suspicion of congenital LQTS, without an alternative explanation, negative or variant of uncertain significance results should be reviewed, this may include reevaluation of existing results, genetic testing, and/or clinical evaluation with ECG and possibly provocative testing.

All of the symptomatic and asymptomatic patients with congenital LQTS should be treated. The first step is the avoidance of medications that have QT-prolonging effects and the management of dehydration, hypokalemia, hypomagnesemia, and high catecholamine levels that can aggravate long QT [10]. Beta-blockers are the choice of treatment in asymptomatic and symptomatic patients with congenital LQTS since they reduce both syncope and SCD [10]. Propranolol, used in our case, provides superior efficacy in this patient population. The use of atenolol and metoprolol has been associated with an increased rate of recurrences [11]. Mexiletine is a QT-attenuating and has a significant protective effect in selected types, which can be used in combination therapy with propranolol [12]. Other treatment modalities, such as left cardiac sympathetic denervation for those with persistent arrhythmias or in patients who cannot tolerate beta blockers and Implantable cardioverter-defibrillator for patients who present with resuscitated SCA or those who have recurrent major events [10].

## Conclusions

Prolonged QT interval is a life-threatening condition that needs immediate intervention. Clinicians should be vigilant to consider this disorder even if there is an associated illness like viral infection which can mimic the illness. ECG is the key part in the diagnosis and follow-up with cardiology is important. Family screening, early diagnosis, management, avoidance of medicines that could cause long QT intervals as well as cardiopulmonary resuscitation training are key to saving a patient’s life.
